# NEDD4L-Mediated Ubiquitination of GPX4 Exacerbates Doxorubicin-Induced Cardiotoxicity

**DOI:** 10.3390/ijms26178201

**Published:** 2025-08-23

**Authors:** Jiaxing Ke, Lingjia Li, Shuling Chen, Chenxin Liao, Feng Peng, Dajun Chai, Jinxiu Lin

**Affiliations:** The Higher Educational Key Laboratory for Cardiovascular Disease of Fujian Province, Clinical Research Center for Metabolic Heart Disease of Fujian Province, Cardiovascular Department, The First Affiliated Hospital, Fujian Medical University, Fuzhou 350005, China; kejiaxing@fjmu.edu.cn (J.K.); youngalee0222@163.com (L.L.); liaochenxin@fjmu.edu.cn (C.L.); pengfeng@fjmu.edu.cn (F.P.); dajunchai-fy@fjmu.edu.cn (D.C.)

**Keywords:** doxorubicin, cardiotoxicity, NEDD4L, GPX4, ubiquitination

## Abstract

Doxorubicin (DOX) is an anthracycline chemotherapeutic agent that is clinically limited by doxorubicin-induced cardiotoxicity (DIC), with ferroptosis and apoptosis identified as key mechanisms. As an antioxidant enzyme, GPX4 undergoes ubiquitin-mediated degradation during myocardial ischemia–reperfusion injury; however, the role of its ubiquitination in DIC remains unclear. This study revealed that GPX4 undergoes ubiquitinated degradation during DIC, exacerbating ferroptosis and apoptosis in cardiomyocytes. NEDD4L was found to interact with GPX4, and its expression was upregulated in DOX-treated mouse myocardial tissues and cardiomyocytes. NEDD4L knockdown alleviated DIC, as well as ferroptosis and apoptosis in cardiomyocytes. Mechanistically, NEDD4L recognizes GPX4 through its WW domain and mediates K48-linked ubiquitination and degradation of GPX4 under DOX stimulation via its HECT domain. Knockdown of NEDD4L reduced DOX-induced GPX4 ubiquitination levels and subsequent degradation. Notably, while NEDD4L knockdown mitigated DOX-induced cell death, concurrent GPX4 knockdown attenuated this protective effect, indicating that GPX4 is a key downstream target of NEDD4L in regulating cardiomyocyte death. These findings identify NEDD4L as a potential therapeutic target for preventing and treating DIC.

## 1. Introduction

Cardio-oncology is an emerging field focused on the intersection of cancer treatment and cardiovascular health [1]. As the use of chemotherapy and targeted therapies has surged, significantly improving cancer survival rates, it has also led to a range of cardiovascular complications collectively referred to as cardiotoxicity [2]. Doxorubicin (DOX), an anthracycline chemotherapeutic agent widely used in treating various cancers, exhibits potent anti-tumor efficacy [3]. However, its dose-dependent cardiotoxicity significantly limits its clinical utility, with severe cases potentially leading to irreversible heart failure [3,4]. Understanding the biological mechanisms underlying DOX-induced cardiotoxicity (DIC) is crucial for developing novel therapeutics to overcome the limitations of conventional chemotherapy.

Oxidative stress is considered a central mechanism in DIC [5]. DOX interacts with mitochondria within cardiomyocytes, catalyzing the generation of a substantial amount of reactive oxygen species (ROS) [5,6]. Due to the inherently weak antioxidant defenses of cardiomyocytes, these cells are particularly susceptible to oxidative stress. ROS trigger lipid peroxidation, protein oxidation, and DNA damage, leading to both ferroptosis and apoptosis in cardiomyocytes [7,8]. Ferroptosis is a form of iron-dependent cell death that is characterized by the accumulation of lipid peroxides [9]. Recent studies have highlighted the crucial role of ferroptosis in DIC [10]. Apoptosis represents another vital mechanism in this process, with DOX activating apoptotic pathways in cardiomyocytes through multiple routes [11].

Glutathione peroxidase 4 (GPX4) is a key enzyme in maintaining cellular antioxidant capacity and membrane stability [12]. GPX4 plays a pivotal role not only in ferroptosis but also in apoptosis, with its antioxidant function serving as a protective factor against both forms of cell death [13,14]. The loss of GPX4 function can concurrently trigger ferroptosis and apoptosis, especially under DOX-induced conditions, in which these two cell death pathways may interact through shared signaling molecules, including mitochondria [11,15]. Given the multifaceted mechanisms of DIC, future therapeutic strategies should consider integrating antioxidant defenses, iron homeostasis regulation, and apoptosis inhibition. The upregulation of GPX4 could serve as an effective intervention target in mitigating DIC.

Additionally, the ubiquitination of GPX4 plays a crucial role in ferroptosis [16,17]. Neural precursor cell expressed developmentally down-regulated protein 4-like (NEDD4L), a member of the NEDD4 family and an important E3 ubiquitin ligase, has been reported to reduce GPX4 stability and activity through ubiquitination [18,19]. This regulatory process is potentially linked to the modulation of intracellular oxidative stress and antioxidant capacity, which is critical for maintaining cellular homeostasis. Recently, the relationship between NEDD4L and cardiovascular diseases has garnered significant attention [20]. However, its role in DIC remains unexplored.

In this study, we elucidate the role of NEDD4L in cardiomyocytes under DOX treatment. DOX promotes the interaction between NEDD4L and GPX4, directly increasing GPX4 ubiquitination and degradation, leading to ferroptosis and apoptosis in cardiomyocytes. Our findings provide novel insights into the pathogenesis of DIC and identify NEDD4L as a potential therapeutic target for mitigating this condition.

## 2. Results

### 2.1. DOX-Induced Ferroptosis and Apoptosis in Cardiomyocytes

To explore the predominant form of cell death induced by DOX in cardiomyocytes, we conducted a Cell Counting Kit-8 (CCK-8) assay. The results indicated that Trolox (an antioxidant), Ferrostatin-1 (Fer-1, a ferroptosis inhibitor), and Z-VAD-FMK (an apoptosis inhibitor) could alleviate DOX-induced cell death (Figure S1A). Western blot analysis further substantiated these observations, demonstrating that DOX reduced the expression of the anti-ferroptotic protein GPX4 (Figure S1B). Additionally, we observed upregulated markers of ferroptosis, including ROS, malondialdehyde (MDA), C11-BODIPY, and ferrous (Fe^2+^) (Figure S1C–F). During DOX intervention in H9C2 cells, apoptotic cell death was also observed, characterized by elevated levels of pro-apoptotic proteins Bax and Cleaved Caspase3 (Figure S1G). Flow cytometry analysis confirmed the induction of apoptosis, as evidenced by an increased proportion of Annexin V^+^/PI^−^ and Annexin V^+^/PI^+^ cells (Figure S1H).

In addition, in vivo experiments revealed that DOX decreased cardiac function, as evidenced by a reduced ejection fraction indicated by echocardiography (Figure S2A–C). To determine the role of ferroptosis in DIC, we evaluated several ferroptosis-related parameters. Compared with the control group, the content of GPX4 in cardiac tissue was significantly reduced (Figure S2D,E), while Fe^2+^ accumulation and MDA production were observed (Figure S2F,G). Furthermore, DOX significantly increased the extent of myocardial apoptosis, as indicated by a marked increase in Bax and Cleaved Caspase3 (Figure S2H) and an increase in the proportion of terminal deoxynucleotidyl transferase–mediated dUTP nick end labeling (TUNEL)-positive cardiomyocytes (Figure S2I).

### 2.2. DOX-Induced Ubiquitin-Mediated Degradation of GPX4

**Figure 1:**
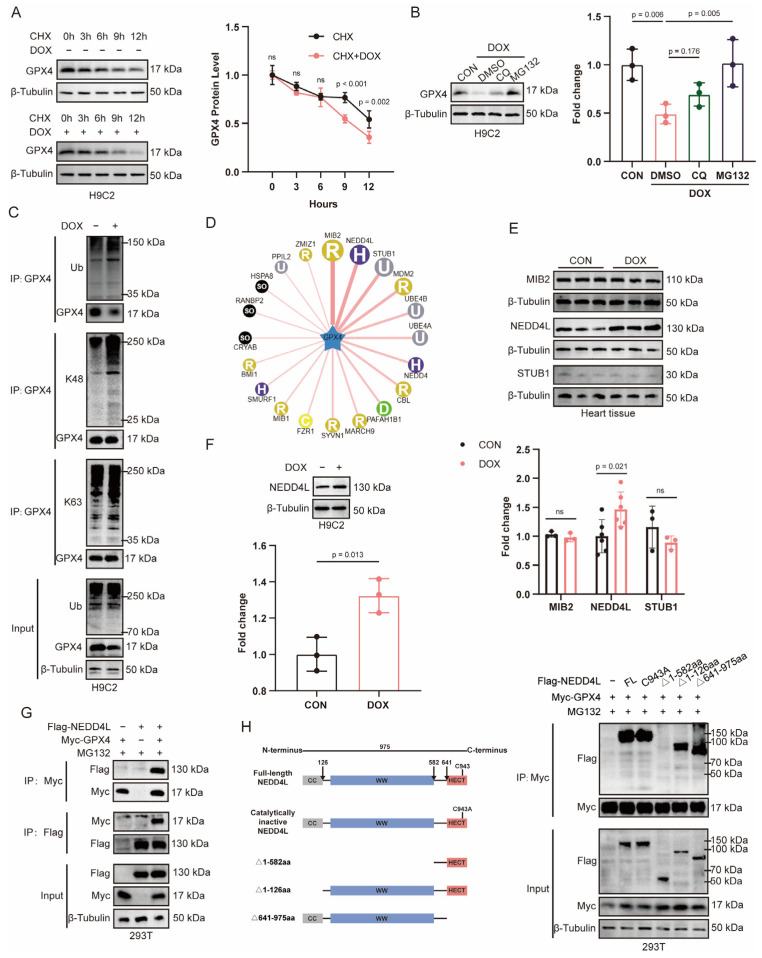
Doxorubicin (DOX) increases NEDD4L expression in cardiomyocytes, and NEDD4L interacts with GPX4. (**A**) Representative immunoblots and relative quantification of GPX4 in H9C2 cells treated with cycloheximide (CHX, 10 μM) at the indicated time points with or without DOX (2 μM) incubation (*n* = 3). ns = not significant. (**B**) Representative immunoblots and relative quantification of GPX4 in H9C2 cells pre-treated with MG132 (10 μM) or chloroquine (CQ, 10 μM) for 30 min prior to DOX (2 μM, 12 h) exposure. (**C**) Immunoprecipitation of total, K48-, and K63-linked polyubiquitination in H9C2 cells with or without DOX (2 μM, 24 h) intervention, utilizing an anti-GPX4 antibody. (**D**) The ubiquitin ligase of GPX4 was predicted using UbiBrowser (http://ubibrowser.ncpsb.org.cn (accessed on 20 August 2025)). (**E**) Immunoblots and relative quantification of MIB2, NEDD4L, and STUB1 in heart tissues from mice subjected to a single intraperitoneal injection of either saline or DOX (15 mg/kg) 7 days post-treatment (*n* = 3–6). (**F**) Representative immunoblots and relative quantification of NEDD4L in H9C2 cells following treatment with DOX (2 μM, 24 h). (**G**) Immunoprecipitation in HEK293T cells transfected with Flag-NEDD4L and Myc-GPX4 using anti-Flag or anti-Myc antibody, followed by immunoblot analysis with the indicated antibodies. (**H**) Immunoprecipitation in HEK293T cells cotransfected with Myc-GPX4 and truncated Flag-NEDD4L or catalytically inactive NEDD4L (C943A) using an anti-Myc antibody, followed by immunoblot analysis with the indicated antibodies.

The inhibition of GPX4 plays an important role in cardiomyocyte death. Published studies indicate that GPX4 ubiquitination is critical for cardiomyocyte survival [16]. We therefore hypothesized that during DOX-induced cardiomyocyte death, GPX4 undergoes ubiquitin-mediated degradation. To test this hypothesis, we used the protein synthesis inhibitor cycloheximide (CHX) to assess GPX4 protein levels in DOX-treated cells. Our results demonstrated that DOX treatment significantly shortened the half-life of GPX4 (Figure 1A). Given that the ubiquitin proteasome system and the autophagy lysosomal pathway are the two primary mechanisms for protein degradation, we treated cells with MG132 (a proteasome inhibitor) or chloroquine (CQ, a lysosome inhibitor) and observed that GPX4 protein levels were sensitive to MG132 but not to CQ (Figure 1B), indicating that the DOX-induced reduction in GPX4 was partially mediated through the proteasome pathway. Consistent with this finding, DOX treatment increased the ubiquitination of GPX4 with K48- and K63-linked ubiquitin chains in cardiomyocytes (Figure 1C).

### 2.3. NEDD4L Was Highly Expressed in DOX-Treated Cardiac Tissue and Cardiomyocytes

Protein ubiquitination, a process primarily orchestrated by E3 ubiquitin ligases, is a critical post-translational modification that plays a significant role in various cellular pathways [21,22]. Using UbiBrowser (http://ubibrowser.ncpsb.org (accessed on 20 August 2025)), we predicted potential ubiquitination-related enzymes that target GPX4 (Figure 1D), and identified three E3 ligases, MIB2, NEDD4L, and STUB1, that are most likely involved in the ubiquitination of GPX4. Further validation through a Western blot analysis revealed a significant upregulation of NEDD4L expression in DOX-treated hearts, while no notable changes were observed for MIB2 and STUB1 (Figure 1E). Immunohistochemical staining of cardiac tissue further confirmed the increased expression of NEDD4L in response to DOX treatment (Figure S3). Similarly, an increase in NEDD4L expression was also noted in H9C2 cells treated with DOX in vitro (Figure 1F).

To investigate whether GPX4 interacts with NEDD4L, we conducted co-immunoprecipitation (co-IP) assays, which notably demonstrated an interaction between GPX4 and NEDD4L. Moreover, this interaction was further substantiated using reverse IP experiments, confirming the binding between NEDD4L and GPX4 (Figure 1G). To further characterize the interaction domains between NEDD4L and GPX4, co-IP assays were performed using truncated forms of NEDD4L and the catalytically inactive mutant (C943A). The WW domain of NEDD4L was identified as essential for its interaction with GPX4 (Figure 1H).

### 2.4. NEDD4L Knockdown Ameliorated Myocardial Dysfunction in DOX-Induced Cardiotoxicity (DIC)

**Figure 2:**
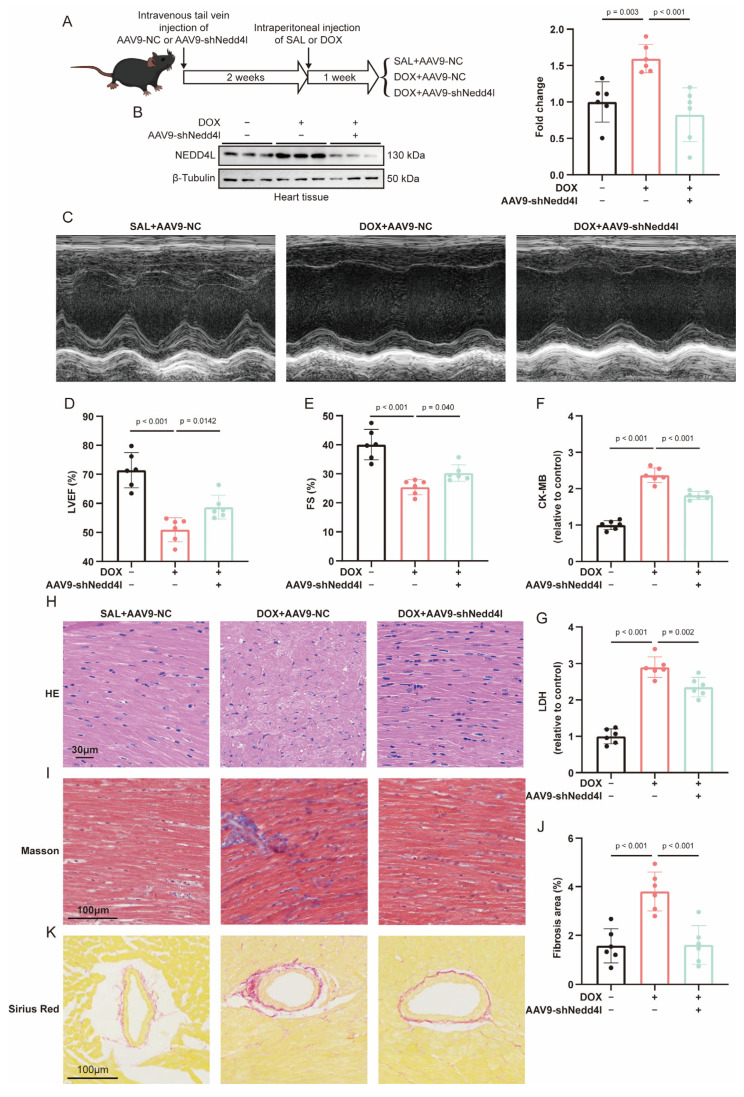
Knockdown of NEDD4L mitigates doxorubicin (DOX)-induced cardiotoxicity (DIC). (**A**) Schematic diagram of the animal experimental protocol. Mice were randomly divided into three groups and underwent tail vein injections of serotype 9 adeno-associated virus (AAV9) carrying either the negative control (NC) or short hairpin RNA (shRNA) targeting Nedd4l to achieve Nedd4l knockdown. Two weeks post-AAV9 injection, DOX (15 mg/kg) was administered intraperitoneally to establish a mouse model of DIC, with saline (SAL) injection serving as a control. The three groups were as follows: SAL+AAV9-NC, DOX+AAV9-NC, and DOX+AAV9-shNedd4l. (**B**) Representative immunoblots and relative quantification of NEDD4L in heart tissue of mice from 3 groups (*n* = 6). (**C**–**E**) Representative left ventricular M-mode echocardiography images (**C**) and echocardiographic assessment of left ventricular ejection fraction (LVEF, (**D**)) and fractional shortening (FS, (**E**)) at 7 days post-intraperitoneal SAL or DOX injection (*n* = 6). (**F**,**G**) Relative quantification of serum creatine kinase-myocardial band (CK-MB, (**F**)) and lactate dehydrogenase (LDH, (**G**)) levels in mice from 3 groups 7 days post-treatment with SAL or DOX, as determined using enzyme-linked immunosorbent assay (ELISA) (*n* = 6). (**F**–**H**) Histological images of hematoxylin and eosin (HE, (**H**)), Masson’s trichrome (**I**), and Sirius red staining (**K**), along with quantification of fibrosis areas ((**J**), *n* = 6), in mice from 3 groups 7 days post-treatment with SAL or DOX.

The precise role of NEDD4L in DIC remains unclear. To explore its potential involvement, we injected mice with two cardiac troponin T (cTnT) promoter-driven serotype 9 adeno-associated virus (AAV9) vectors: one encoding a short hairpin RNA (shRNA) targeting Nedd4l (AAV9-shNedd4l) to establish a cardiomyocyte-specific NEDD4L knockdown model and the other being a negative control vector (AAV9-NC). Two weeks after the viral injection, the mice received intraperitoneal DOX or saline (SAL), establishing three experimental groups: SAL+AAV9-NC, DOX+AAV9-NC, and DOX+AAV9-shNedd4l (Figure 2A). A Western blot analysis confirmed the efficient knockdown of NEDD4L in cardiac tissue (Figure 2B).

Cardiac function was evaluated using echocardiography, revealing that, compared with the DOX+AAV9-NC group, the NEDD4L-knockdown DIC group (DOX+AAV9-shNedd4l) exhibited significant improvements post-DOX injection, including an increased left ventricular ejection fraction (LVEF) and fractional shortening (FS) (Figure 2C–E).

Further analysis of myocardial injury biomarkers such as creatine kinase-myocardial band (CK-MB) and lactate dehydrogenase (LDH) demonstrated that NEDD4L knockdown significantly mitigated DOX-induced myocardial injury (Figure 2F,G). Furthermore, hematoxylin and eosin (HE) staining demonstrated that the knockdown of NEDD4L mitigated the structural disarray in cardiac tissue induced using DOX treatment (Figure 2H). Cardiac fibrosis was assessed using Masson’s trichrome and Sirius red staining, revealing a pronounced enhancement in fibrotic areas post-DOX treatment. Notably, this fibrotic response was substantially attenuated in the NEDD4L knockdown group (Figure 2I–K).

### 2.5. Knockdown of NEDD4L Suppressed DOX-Induced Ferroptosis and Apoptosis In Vivo

**Figure 3:**
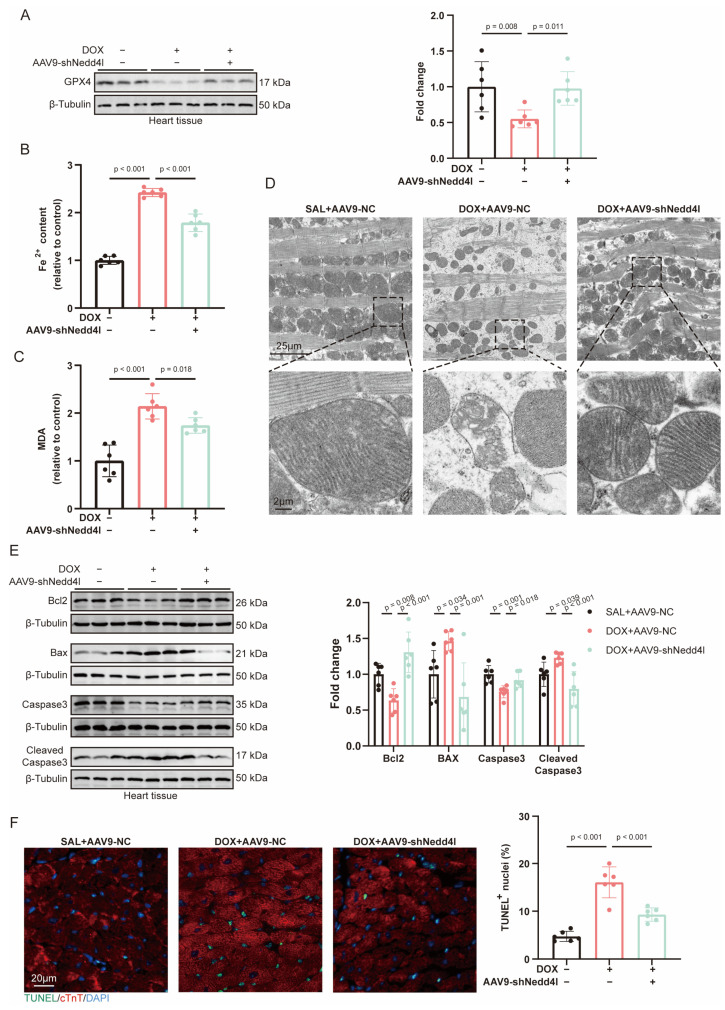
NEDD4L knockdown prevented doxorubicin (DOX)-induced ferroptosis and apoptosis in vivo. (**A**) Representative immunoblots and relative quantification of GPX4 in cardiac tissue from mice of SAL+AAV9-NC, DOX+AAV9-NC, and DOX+AAV9-shNedd4l groups 7 days post-treatment with saline (SAL) or DOX (15 mg/kg) (*n* = 6). (**B**,**C**) Relative quantification of myocardial iron content (Fe^2+^, (**B**)) and malondialdehyde (MDA, (**C**)) levels in mice from 3 groups (*n* = 6). (**D**) Transmission electron microscopy (TEM) images of mitochondria in heart tissue of mice from 3 groups. (**E**) Representative immunoblots and relative quantification of proteins related to apoptosis in heart tissues of mice from 3 groups (*n* = 6). (**F**) Representative images of terminal deoxynucleotidyl transferase-mediated dUTP nick end labeling (TUNEL) staining and quantification of TUNEL-positive nuclei in heart tissues of mice from 3 groups (*n* = 6).

As observed in our previous results, GPX4 expression was significantly downregulated following DOX treatment. However, knockdown of NEDD4L partially restored GPX4 levels (Figure 3A). Ferroptosis-related markers, such as Fe^2+^ and MDA levels, were elevated under DOX treatment. Knockdown of NEDD4L effectively mitigated these changes (Figure 3B,C). Transmission electron microscopy (TEM) revealed mitochondrial abnormalities after DOX treatment, including increased electron density, cristae disorder, and membrane damage. Notably, NEDD4L knockdown partially reversed these mitochondrial structural abnormalities (Figure 3D).

Regarding apoptosis, NEDD4L knockdown alleviated the DOX-induced decrease in anti-apoptotic proteins Bcl2 and Caspase3, as well as the increase in pro-apoptotic proteins Bax and Cleaved Caspase3 in myocardial tissue (Figure 3E). Similarly, the number of TUNEL-positive myocardial cells induced by DOX was reduced after NEDD4L knockdown (Figure 3F).

### 2.6. Knockdown of NEDD4L Alleviated DOX-Induced Ferroptosis and Apoptosis In Vitro

To evaluate the influence of NEDD4L on DIC in vitro, H9C2 cells were transfected with small interfering RNA (siRNA) targeting NEDD4L (siNedd4l) to knock down NEDD4L expression. Western blot analysis confirmed knockdown efficiency, and among the siRNAs, siNedd4l-2 exhibited a significantly higher efficacy, prompting its selection for subsequent experiments (Figure S4A). siNedd4l-2 effectively suppressed the DOX-induced upregulation of NEDD4L expression (Figure S4B).

**Figure 4:**
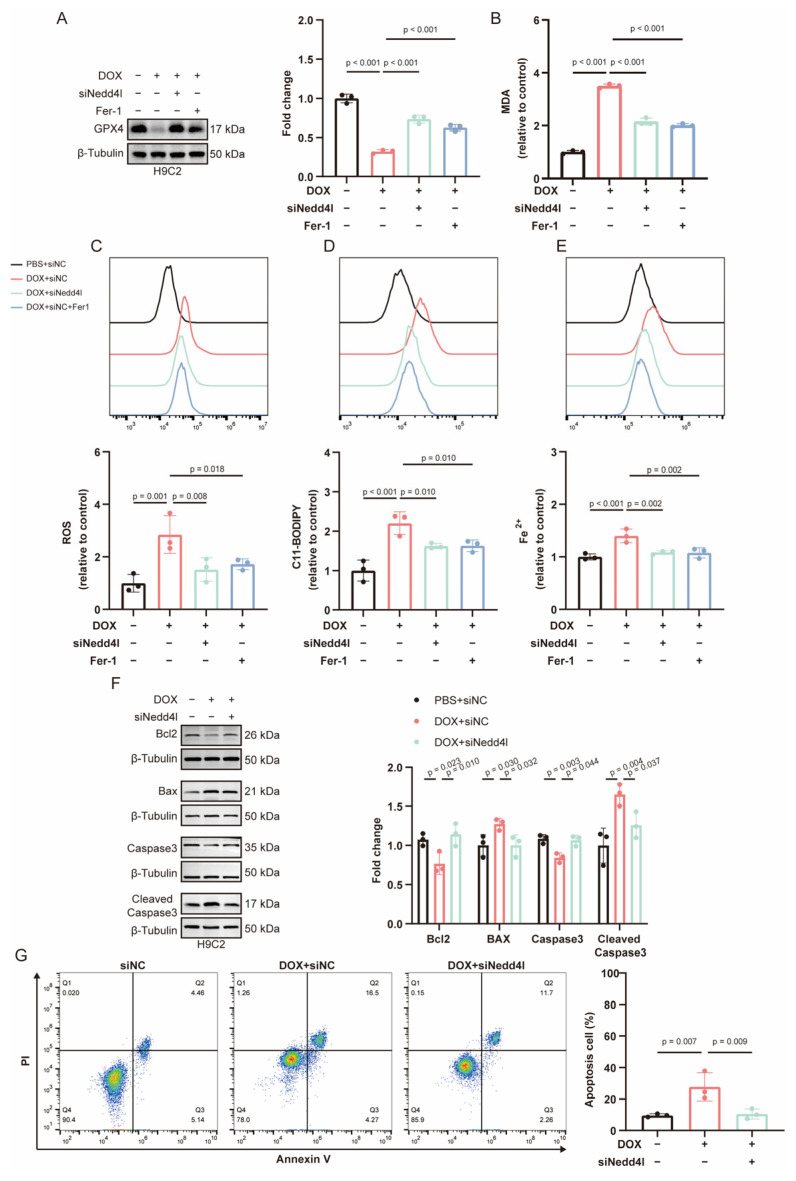
NEDD4L knockdown inhibits doxorubicin (DOX)-induced ferroptosis and apoptosis in vitro. (**A**) Representative immunoblots and relative quantification of GPX4 protein levels in H9C2 cells following treatment with small interfering RNA (siRNA) targeting NEDD4L (siNedd4l) or a non-targeting control (siNC), exposure to DOX, or combined treatment with DOX and Ferrostatin-1 (Fer-1) for 24 h (*n* = 3). (**B**) The malondialdehyde (MDA) content of H9C2 cells following treatment with siNedd4l or siNC, exposure to DOX, or combined treatment with DOX and Fer-1 for 24 h (*n* = 3). (**C**–**E**) Histogram and relative quantification of reactive oxygen species (ROS, (**C**)), lipid peroxide (**D**), and ferrous iron (Fe^2+^, (**E**)) levels in H9C2 cells, detected using the staining probe of DCFH-DA, C11-BODIPY, and FerroOrange, respectively. The cells were treated with siNedd4l or siNC, subjected to DOX, or co-treated with DOX and Fer-1 for 24 h (*n* = 3). (**F**) Representative immunoblots and relative quantification of proteins related to apoptosis in H9C2 cells treated with siNedd4l or siNC, subjected to DOX for 24 h (*n* = 3). (**G**) Effects of NEDD4L knockdown and DOX treatment on cardiomyocyte apoptosis determined using flow cytometry analysis (*n* =3).

Treatment with DOX led to a significant reduction in GPX4 levels in H9C2 cells. NEDD4L knockdown effectively counteracted this alteration in GPX4 protein expression. Notably, the effect of Fer-1 on GPX4 protein levels was comparable to that observed with NEDD4L knockdown (Figure 4A). In the DOX treatment group, MDA levels were significantly increased; however, MDA levels were restored after NEDD4L knockdown or Fer-1 intervention (Figure 4B). Subsequently, we further examined other markers of ferroptosis. The DOX-induced increase in ferroptosis-related markers, including ROS, lipid ROS, and Fe^2+^, was attenuated by the NEDD4L knockdown or Fer-1 intervention (Figure 4C–E).

Moreover, DOX-induced increases in Bax and Cleaved Caspase3 in cardiomyocytes were downregulated following NEDD4L knockdown, whereas the DOX-induced decreases in Bcl2 and Caspase3 were partially reversed (Figure 4F). Flow cytometry analysis also demonstrated a significant increase in apoptosis post-DOX treatment, which was reduced by NEDD4L knockdown (Figure 4G).

### 2.7. NEDD4L Promotes Proteasomal Degradation of GPX4 via K48-Linked Polyubiquitination Following DOX Treatment

**Figure 5:**
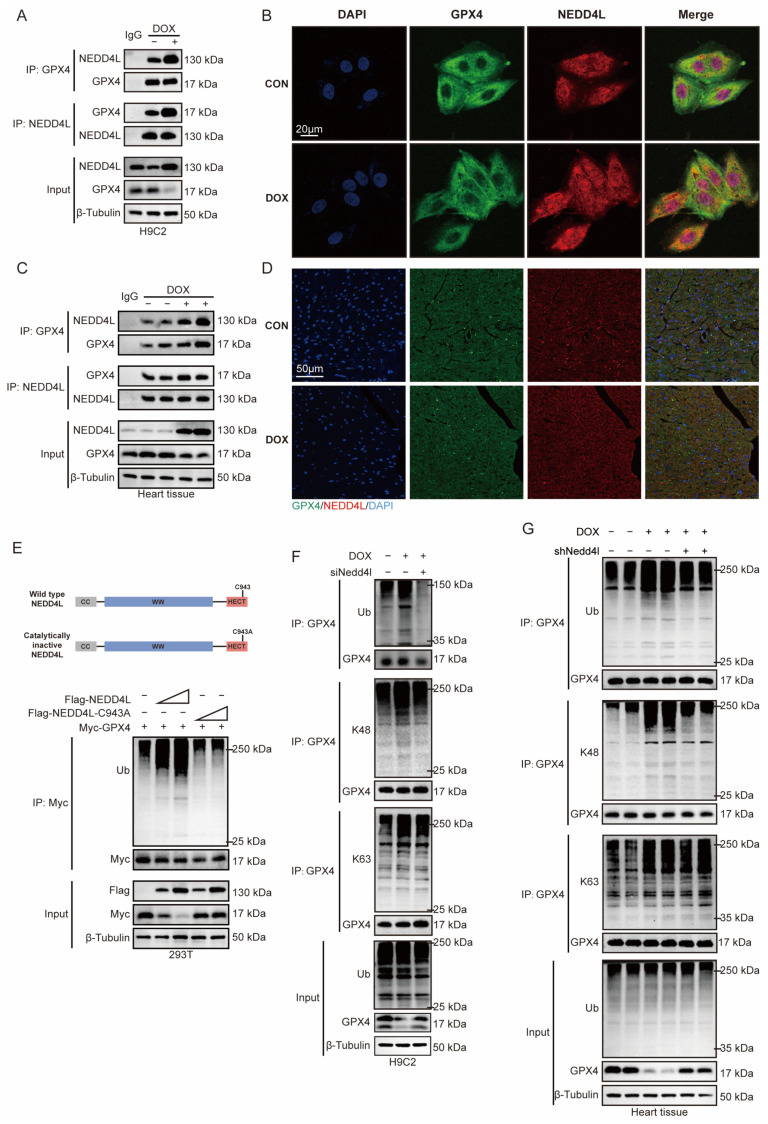
Doxorubicin (DOX) enhances the interaction between NEDD4L and GPX4, thereby promoting proteasomal degradation of GPX4 via K48-linked polyubiquitination. (**A**) Immunocoprecipitation of NEDD4L and GPX4 in H9C2 cells incubated with (2 μM, 24 h) or without DOX, using antibodies specific to GPX4 and NEDD4L or a non-specific control IgG, after which the precipitated proteins were analyzed using Western blot analysis with antibodies recognizing GPX4 and NEDD4L. (**B**) Colocalization of GPX4 (green) with NEDD4L (red) in H9C2 cells in the presence or absence of DOX treatment (2 μM, 24 h). (**C**) Immunocoprecipitation of NEDD4L and GPX4 in mouse heart tissue following a single intraperitoneal injection of either saline (SAL) or DOX (15 mg/kg) 7 days post-injection, using GPX4/NEDD4L-specific antibody or control IgG, followed by probing with antibodies specific for GPX4/NEDD4L. (**D**) Colocalization of GPX4 (green) with NEDD4L (red) in the mouse heart tissue following a single intraperitoneal injection of either SAL or DOX (15 mg/kg) 7 days post-injection. (**E**) Immunocoprecipitation of GPX4 ubiquitination in HEK293T cells transfected with Myc-GPX4, and increasing amounts of Flag-NEDD4L or C943A, utilizing an anti-GPX4 antibody. (**F**) Immunoprecipitation of total, K48-, and K63-linked polyubiquitination in H9C2 cells pretreated with or without siNEDD4L intervention, and subsequently treated with or without DOX (2 μM, 24 h) intervention, utilizing an anti-GPX4 antibody. (**G**) Immunoprecipitation of total, K48-, and K63-linked polyubiquitination in heart tissue of mice from mice of SAL+AAV9-NC, DOX+AAV9-NC, and DOX+AAV9-shNedd4l groups, utilizing an anti-GPX4 antibody.

We previously demonstrated that GPX4 and NEDD4L can form a complex with each other. Through co-IP and immunofluorescence assays, we validated the interaction between NEDD4L and GPX4 and confirmed the complex formation both in vitro and in vivo. Moreover, we found that DOX can enhance this binding interaction (Figure 5A–D).

To further investigate whether NEDD4L affects the protein stability of GPX4, we transfected HEK293T cells with NEDD4L or its catalytically inactive mutant, C943A. NEDD4L reduced GPX4 protein levels and increased GPX4 ubiquitination in a dose-dependent manner, whereas the NEDD4L C943A mutant had no effect on GPX4 (Figure 5E), indicating that the enzymatic activity of NEDD4L is essential for regulating GPX4 stability.

To explore whether endogenous NEDD4L is involved in the DOX-induced degradation of GPX4, we transfected H9C2 cells with siNedd4l. Consistent with our previous findings, DOX reduced GPX4 protein levels and increased GPX4 ubiquitination, while NEDD4L silencing increased GPX4 protein levels. We also observed that NEDD4L knockdown decreased K48-linked ubiquitination of GPX4, whereas K63-linked ubiquitination remained unchanged (Figure 5F). Similarly, in vivo experiments using the AAV9-mediated knockdown of NEDD4L in cardiomyocytes reversed the DOX-induced reduction in GPX4 protein levels and alleviated the DOX-induced K48-linked ubiquitination of GPX4 (Figure 5G).

### 2.8. NEDD4L Regulates GPX4 to Promote Ferroptosis and Apoptosis

To downregulate Gpx4 expression in H9C2 cells, we employed siGpx4 and identified siGpx4-1 as the most effective siRNA via Western blot analysis (Figure S5A), which was thus selected for subsequent experiments. To establish that NEDD4L regulates GPX4 to promote ferroptosis and apoptosis in DIC, we simultaneously knocked down NEDD4L and GPX4 in H9C2 cells. Under DOX treatment, silencing NEDD4L restored GPX4 protein levels, whereas the simultaneous knockdown of both genes resulted in a significant reduction in GPX4 (Figure S5B).

**Figure 6:**
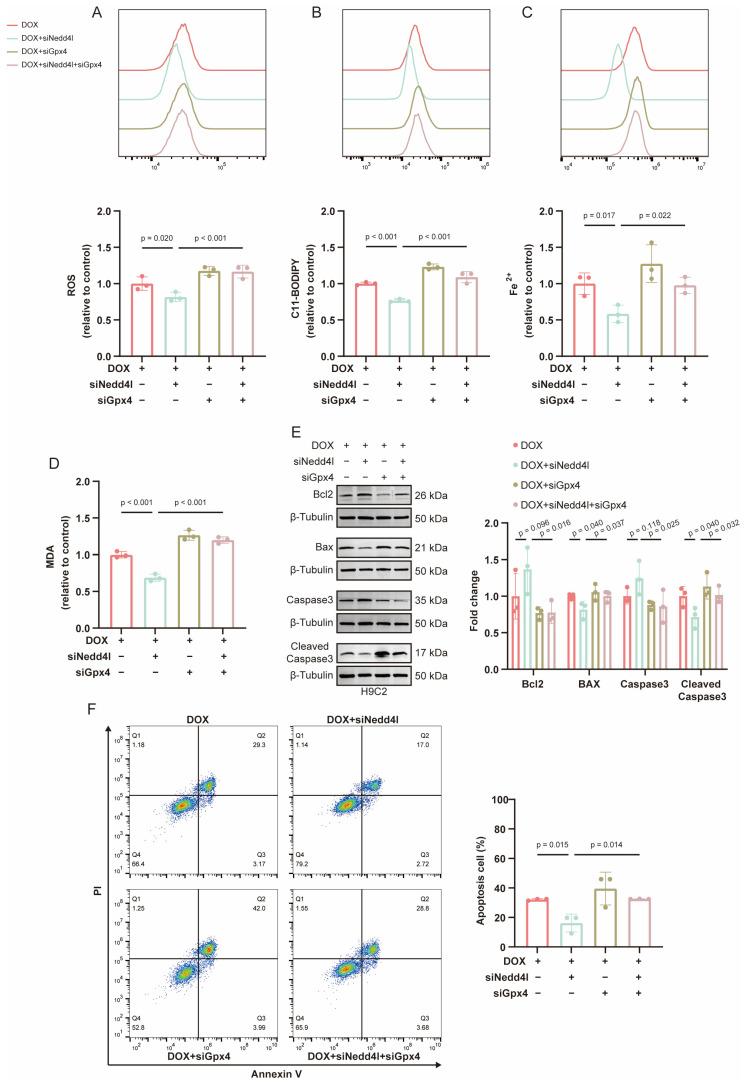
NEDD4L facilitates ferroptosis and apoptosis in cardiomyocytes by suppressing GPX4 in doxorubicin (DOX)-induced cardiotoxicity (DIC). (**A**–**C**) Histogram and relative quantification of reactive oxygen species (ROS, **A**), lipid peroxide (**B**), and ferrous iron (**C**) levels in H9C2 cells, detected using the staining probe of DCFH-DA, C11-BODIPY, and FerroOrange, respectively. The cells were pre-treated with either small interfering RNA targeting NEDD4L (siNedd4l) or a non-targeting control (siNC), as well as with siGpx4 or siNC, followed by treatment with DOX (2 μM, 24 h, *n* = 3). (**D**) The malondialdehyde (MDA) content of H9C2 cells with siNedd4l/siNC and siGpx4/siNC pretreatments followed by DOX intervention (*n* = 3). (**E**) Representative immunoblots and relative quantification of proteins related to apoptosis in H9C2 cells with siNedd4l/siNC and siGpx4/siNC pretreatments followed by DOX intervention (*n* = 3). (**F**) Effects of NEDD4L knockdown, GPX4 knockdown, and DOX treatment on cardiomyocyte apoptosis determined using flow cytometry analysis (*n* = 3).

Functionally, DOX elicited ferroptosis, as evidenced by increased C11-BODIPY oxidation, elevated labile Fe^2+^, and higher ROS and MDA levels. These alterations were reversed by NEDD4L knockdown. In contrast, GPX4 silencing with siGpx4 reinstated ferroptotic readouts in DOX-treated cells and abolished the protection conferred by NEDD4L knockdown (Figure 6A–D).

Apoptosis analyses were concordant. DOX reduced Bcl2 and Caspase3, increased Bax and Cleaved Caspase3, and augmented the proportion of Annexin V/PI-positive cells. NEDD4L knockdown attenuated these changes, whereas concurrent GPX4 silencing significantly increased apoptosis irrespective of NEDD4L knockdown (Figure 6E,F).

Collectively, these findings indicate that NEDD4L promotes DOX-induced ferroptosis and apoptosis through the suppression of GPX4. The preservation of GPX4 via NEDD4L depletion is protective, an effect that is abrogated when GPX4 is concomitantly silenced.

## 3. Discussion

DOX is an effective chemotherapeutic agent that is widely used in treating various malignancies [23]. However, its clinical application is significantly limited by its cardiotoxic side effects, for which no effective pharmacological interventions are currently available [24,25]. Our study identified NEDD4L as a crucial promoter of ferroptosis and apoptosis in cardiomyocytes during DIC. Mechanistically, we found that NEDD4L expression was significantly upregulated during DOX-induced cardiomyocyte death. NEDD4L facilitated the ubiquitination and subsequent degradation of GPX4, leading to enhanced ferroptosis and apoptosis in cardiomyocytes. Knockdown of NEDD4L in cardiomyocytes was shown to inhibit ferroptosis and apoptosis, thereby mitigating DOX-induced cardiac injury in mice. Therefore, targeting NEDD4L may represent a novel therapeutic strategy for treating DIC.

Previous studies have implicated several pathological processes in DIC, including increased ROS production, mitochondrial dysfunction, and increased cardiac cell death [3,4,5]. The modes of cell death induced by DOX are diverse, encompassing apoptosis, necrosis, ferroptosis, and other forms of cell death. Recent research has emphasized the critical role of ferroptosis in DIC, showing that inhibition of ferroptosis with Fer-1 can significantly reduce DOX-induced cardiac injury [10,24,26]. Additionally, the role of cardiomyocyte apoptosis in DIC has been well documented, consistent with our experimental findings [3,13,27]. Therefore, ferroptosis and apoptosis may represent the primary modes of cell death in the context of DIC.

GPX4 is known to play an essential role in oxidative stress, ferroptosis, and apoptosis [12,13,14]. As a member of the glutathione peroxidase family, GPX4 primarily functions to protect cellular membranes from oxidative damage. GPX4 activity depends on glutathione (GSH) as a reducing agent, converting toxic lipid peroxides into non-toxic lipid alcohols, thereby mitigating oxidative stress and inhibiting cell death [28]. Studies have shown that GPX4 expression is downregulated in DIC models, directly leading to increased ferroptosis in cardiomyocytes and exacerbating cardiac injury [28,29,30]. The upregulation of GPX4 expression or enhancement of its activity through exogenous antioxidant administration, such as N-Acetyl-L-Cysteine (NAC), has been demonstrated to effectively reduce DIC [31]. This protective effect is primarily attributed to the ability of GPX4 to clear lipid peroxides, thereby inhibiting ferroptosis. Furthermore, GPX4 upregulation can alleviate mitochondrial dysfunction, suppress inflammatory responses, and reduce cardiomyocyte apoptosis, offering additional cardioprotection. In line with these observations, antioxidant strategies may complement approaches that preserve GPX4. A recent study showed that lutein protects against DOX-induced vasculopathy, accompanied by increases in GSH, superoxide dismutase (SOD), and glutathione S-transferase (GST) [32]. Given that GPX4 is a selenoprotein that requires GSH to reduce hydroperoxides, interventions that augment intracellular GSH and bolster endogenous antioxidant enzymes could maintain GPX4 catalytic capacity, limit lipid peroxidation, and thereby restrain ferroptosis in DIC. Such antioxidant support may act additively with strategies that prevent GPX4 degradation.

It has been reported that GPX4 is transcriptionally regulated by NRF2 during DIC, yet whether GPX4 is regulated by other mechanisms remains unclear [30,33,34]. Ubiquitination plays a significant role in cardiovascular diseases, acting as a crucial post-translational modification that regulates protein stability [21,35,36]. Ubiquitination, primarily mediated by E3 ubiquitin ligases in conjunction with E1 and E2 enzymes, promotes proteasome-mediated protein degradation. Previous studies have linked the ubiquitination of GPX4 with E3 ubiquitin ligases such as MIB2, NEDD4L, and TRIM26 [18,19,37,38]. For instance, in myocardial ischemia–reperfusion injury, GPX4 was reported to undergo ubiquitination and subsequent degradation [16]. This raised the question of whether GPX4 is similarly regulated by ubiquitination in DIC. Indeed, our findings confirmed that DOX promotes the ubiquitination and degradation of GPX4.

To identify which ubiquitin ligase promotes GPX4 ubiquitination in DIC, we utilized the UbiBrowser prediction tool and validated the top three candidate ubiquitin ligases. NEDD4L was identified as being upregulated in DIC. NEDD4L is an E3 ligase with an HECT domain that is involved in the pathogenesis of cardiovascular diseases through multiple pathways [20]. For example, the overexpression of circNfix in cardiomyocytes has been reported to promote the interaction between NEDD4L and YBX1, leading to YBX1 ubiquitination-dependent degradation, thereby promoting cardiomyocyte apoptosis and inhibiting proliferation [39]. Similarly, studies have shown that elevated miR-454 targets NEDD4L, reducing NEDD4L-induced TrkA ubiquitination, which subsequently activates the cAMP pathway to mitigate H9C2 cell apoptosis and oxidative stress damage [40]. These findings suggest that NEDD4L regulates various cellular activities, including oxidative stress and cell death; however, the role of NEDD4L in DIC remains unclear. A further investigation revealed that NEDD4L knockdown in cardiomyocytes significantly attenuated DOX-induced ferroptosis and apoptosis, as well as subsequent cardiac dysfunction, suggesting that NEDD4L represents a promising therapeutic target for DIC. Further experimental results showed that GPX4 knockdown in NEDD4L-deficient DIC cardiomyocytes enhanced ferroptosis and apoptosis, indicating that GPX4 upregulation is a major mechanism by which NEDD4L knockdown improves cardiomyocyte survival.

Although our findings do not completely exclude the involvement of other putative NEDD4L-binding proteins, they highlight the critical role of GPX4 as the predominant substrate of NEDD4L in regulating cardiomyocyte survival, particularly in the context of DIC. Recent studies have reported that in a mouse model of heart ischemia–reperfusion, NEDD4L interacts with ACSL4, promoting ACSL4 ubiquitination and degradation, thereby alleviating cardiomyocyte ferroptosis [41]. The discrepancy between our results and the aforementioned studies maybe due to differences in disease models. Whether ACSL4 and GPX4 compete for binding with NEDD4L remains unknown and warrants further investigation. Subsequent research should aim to identify additional NEDD4L substrate proteins and elucidate their contributions to cardiomyocyte survival, thereby enhancing our comprehensive grasp of the underlying mechanisms.

Furthermore, our study also discovered that DOX promotes both K48- and K63-linked ubiquitination of GPX4. However, upon NEDD4L knockdown, only K48-linked ubiquitination was significantly reduced, while K63-linked ubiquitination appeared unchanged. This suggests that other ubiquitin ligases may promote GPX4 ubiquitination during DIC, which represents a future direction for further exploration.

Additionally, protein ubiquitination is a dynamic and reversible process that is regulated by the interplay between E3 ubiquitin ligases and deubiquitinating enzymes (DUBs). In this study, we identified the E3 ubiquitin ligases for GPX4, but did not investigate the potential DUBs involved. This represents a significant gap in our current understanding that should be addressed in future studies to provide more comprehensive insights into the regulatory mechanisms governing cardiomyocyte survival.

Finally, DIC is network-driven, involving crosstalk among iron metabolism, lipid peroxidation, mitochondrial quality control, redox balance, DNA damage signaling, apoptosis, and autophagy. Consistent with systems-level analyses, the NEDD4L–GPX4 axis likely intersects this network, supporting combination strategies that pair antioxidant augmentation with the modulation of ubiquitin-dependent GPX4 turnover [42].

## 4. Materials and Methods

### 4.1. Reagents

DOX (HY-15142), the antioxidant Trolox (HY-101445), the apoptosis inhibitor Z-VAD-FMK (HY-16658B), the ferroptosis inhibitor Fer-1(HY-100579), CHX (HY-12320), the proteasome inhibitor MG132 (HY-13259), and the autophagy inhibitor CQ (HY-17589) were purchased from MCE (Monmouth Junction, NJ, USA). CCK-8(K1018) was purchased from APExBIO (Houston, TX, USA). The GPX4 antibody (R381958) was purchased from Zhengneng (Wuhan, China). Antibodies against Bcl2 (YM8319), Bax (YM8175), Caspase3 (YT0656), and Cleaved Caspase3 (YC0006) were purchased from Immunoway (Plano, TX, USA). Antibodies against ubiquitin (Ub, 43124), Flag (70586), Myc (2276), and DAPI (4083S) were purchased from Cell Signaling Technology (Danvers, MA, USA). Antibodies against K48-linkage-specific polyubiquitin (A3606) were obtained from Abclonal (Wuhan, China). Antibodies against K63-linkage-specific polyubiquitin (ab179434) were obtained from Abcam (Cambridge, UK). The NEDD4L (13690-1-AP) and β-Tubulin (66240-1-Ig) antibodies were purchased from Proteintech (Wuhan, China). The lipid peroxidation detection kit (BODIPY™ 581/591 C11, D3861) was purchased from Thermo Fisher Scientific (Waltham, MA, USA). The ferrous ion detection kit (FerroOrange, F374) was purchased from Dojindo (Kumamoto, Japan). The ROS (S0033S) and MDA (S0131S) detection kits were purchased from Beyotime (Shanghai, China). The Annexin V-FITC/PI apoptosis detection kit (AT101) was purchased from Lianke (Hangzhou, China). The CK-MB (E-EL-M0355), LDH (E-EL-M0419), and Iron Assay (E-BC-K773-M) kits were purchased from Elabscience (Wuhan, China). The cTnT (SAB5702554) antibody was purchased from Sigma-Aldrich (St. Louis, MO, USA).

### 4.2. Cell Culture and Treatment

H9C2 and HEK293T cells were cultured in Dulbecco’s Modified Eagle Medium (DMEM, Gibco, Waltham, MA USA) supplemented with 10% fetal bovine serum (FBS, Gibco), 100 U/mL penicillin, and 100 μg/mL streptomycin at 37 °C in a humidified atmosphere containing 5% CO_2_ and 95% air. H9C2 cells were treated with DOX (2 μM) for 24 h for further analysis.

### 4.3. Cell Viability Assay

Cells were seeded into a 96-well plate at a density of 5 × 10^3^ cells per well and incubated for 24 h with five replicates per group. Thirty minutes prior to DOX treatment, cells were pretreated with the following inhibitors: Trolox (100 μM), Fer-1 (2 μM), Z-VAD-FMK (10 μM), or Baf-A1 (1 nM). Subsequently, cells were treated with DOX (2 μM) for 24 h. After aspiration of the culture supernatant, 100 μL of complete growth medium containing 10% CCK-8 reagent was added to each well. The plates were incubated at 37 °C in a humidified atmosphere with 5% CO_2_ for 2 h, following which the absorbance at a wavelength of 450 nm was determined using a microplate spectrophotometer.

### 4.4. Transfection of Small Interfering RNA (siRNA) and Plasmid

Full-length cDNAs of GPX4 and NEDD4L, together with NEDD4L truncation mutants and the catalytically inactive mutant (C943A), were amplified using standard PCR and subcloned into the GV658 vector to generate Myc- or Flag-tagged constructs; empty GV658 vector served as a control. All plasmids were synthesized by GeneChem Co., Ltd. (Shanghai, China). siRNAs were also obtained from GeneChem. HEK293T cells were used for plasmid overexpression and H9C2 cells for siRNA-mediated knockdown. Transfections were performed with Lipofectamine 2000 (Thermo Fisher Scientific, Waltham, MA, USA). Plasmids or siRNAs were diluted in Opti-MEM (Gibco), complexed with Lipofectamine, and then added to the cells.

The specific siRNA sequences utilized in this study were as follows:

siNedd4l-1, CAGCCAGGUGUUAUGUGGAGAGAAU;

siNedd4l-2, CAGGUGUUAUGUGGAGAGAAUGAUA;

siGpx4-1, GCGUGUGCAUCGUCACCAATT;

siGpx4-2, CCGAGUGUGGUUUACGAAUTT.

### 4.5. Ferrous Iron, Lipid Peroxidation, and ROS Detection

The cells were first rinsed twice with serum-free DMEM or phosphate-buffered saline (PBS), then incubated with FerroOrange (1 μM), C11-BODIPY 581/591 (5 μM), or DCFH-DA (5 μM) at 37 °C in a cell culture incubator with a gas mixture of 95% air and 5% CO_2_ for 20–30 min. Following incubation, the cells were rinsed twice with serum-free medium or PBS, and the fluorescence intensity was measured using a flow cytometer.

### 4.6. Malondialdehyde (MDA) Detection

Determination of MDA levels in H9C2 cells and heart tissue was conducted utilizing the lipid peroxidation MDA assay kit, according to the manufacturer’s protocol, with absorbance measurements at 532 nm. For H9C2 cells, proteins were extracted and quantified using a BCA assay kit (P0011, Beyotime). The MDA concentration was normalized to the corresponding protein concentration. In the case of heart tissue, equal weights of the tissue were processed for protein extraction, and the MDA concentration was normalized to the corresponding tissue mass.

### 4.7. Apoptosis Detection

The cells were harvested and rinsed twice with precooled PBS, and then resuspended in 1× binding buffer. Subsequently, the cells were stained with Annexin V-FITC (5 μM) and propidium iodide (PI, 10 μM) at room temperature for 5 min prior to analysis using flow cytometry. Cells with Annexin V^+^/PI^−^ and Annexin V^+^/PI^+^ were categorized as apoptotic.

### 4.8. Animals

Male C57BL/6 mice (6–8 weeks old) were obtained from the Shanghai Laboratory Animal Center, Chinese Academy of Sciences. This study was conducted in strict accordance with the guidelines set by the Animal Experimental Ethics Committee of Fujian Medical University (IACUC FJMU 2024-0349). Mice were housed at 25 °C with a 12 h light/dark cycle and provided with sterilized food and water ad libitum. After a one-week acclimation period, mice were injected intraperitoneally with doxorubicin (15 mg/kg) to induce DIC, with saline serving as a control. Cardiac function was assessed using echocardiography on day 7 post-treatment. Subsequently, mice were euthanized, and ocular blood was collected for further analysis of cardiac injury markers. Hearts were excised for protein expression, histopathological analysis, and TEM.

### 4.9. Adenovirus and Adeno-Associated Virus

The mice were infected with AAV9 carrying shRNA targeting Nedd4l (AAV9-shNedd4l) or a negative control (AAV9-NC), both under the control of the cTnT promoter. The AAV9 was provided by Bio-Wit Technology Co., Ltd. (Wuhan, China). The sense strand of shNEDD4L is GGTAGTCGGGACTTGTCAGAT. AAV9 (AAV9-NC and AAV9-shNedd4l, 2 × 10^12^ particles) was administered via tail vein injection for in vivo transduction of cardiomyocytes. Subsequently, two weeks post-AAV injection, DOX was administered intraperitoneally. The mice were used for further experiments one week later.

### 4.10. Echocardiography 

The mice were initially anesthetized with 1–2% isoflurane and secured for transthoracic echocardiography using an ultrasound imaging system (Vevo 2100 Imaging System, FUJIFILM VisualSonics, Toronto, ON, Canada or MyLabOmegaVet Imaging System 24.00.13, Esaote, Shenzhen, China). Body temperature was maintained within the physiological range of 35.5–37.5 °C. M-mode recordings were taken at the mid-papillary level of the LVEF, and FS values were measured from the M-mode tracings.

### 4.11. Histological Analysis

Cardiac tissues from mice were embedded in paraffin and sectioned into 5 μm slices for histological evaluation. Deparaffinized myocardial sections were cleared with xylene and dehydrated through a graded ethanol series. Tissue slices were stained with HE, Masson’s trichrome, and Sirius red to assess histological changes.

### 4.12. Immunohistochemistry (IHC)

Paraffin-embedded sections were baked at 60 °C, deparaffinized in xylene, rehydrated through graded ethanol, and subjected to antigen retrieval in citrate buffer. Tissue areas were circumscribed with a hydrophobic barrier pen, endogenous peroxidase activity was quenched, and nonspecific binding was blocked with 5% bovine serum albumin (BSA). Sections were then incubated overnight with primary antibodies against GPX4 (1:200) or NEDD4L (1:200). After they were washed, sections were incubated with a secondary antibody (1:500) at room temperature and then with a streptavidin–peroxidase conjugate. The signal was developed with DAB, and nuclei were counterstained with hematoxylin. Slides were dehydrated through graded ethanol, cleared in xylene, and mounted with neutral resin. Stained sections were visualized under a light microscope.

### 4.13. Terminal Deoxynucleotidyl Transferase-Mediated dUTP In Situ Nick End Labeling (TUNEL) Assay

For the assessment of apoptosis, fresh frozen sections of 5-micrometer thickness were labeled using an in situ cell death detection kit according to the manufacturer’s instructions. The sections were permeabilized with 0.1% Triton X-100 for 10 min and blocked with BSA for 1 h. cTnT primary antibody (1:200) was applied to mark cardiomyocytes. Subsequently, the samples were incubated with a fluorophore-conjugated secondary antibody for 2 h at room temperature. After myocyte staining, the sections were incubated with a fluorescence TUNEL system to detect apoptotic nuclei. Finally, the cell nuclei were counterstained with 4′,6-diamidino-2-phenylindole (DAPI). TUNEL-positive cells were observed under a fluorescence microscope, and the ratio of TUNEL-positive cells to DAPI-positive nuclei was calculated.

### 4.14. Transmission Electron Microscopy (TEM)

Upon euthanasia, the hearts of mice were perfused with PBS to remove blood components. Ventricular myocardial tissue was cut into 1 mm^3^ blocks, fixed in electron microscopy fixative (G1102, Servicebio, Wuhan, China), and then stored at 4 °C. The tissues were post-fixed in 1% osmium tetroxide for 2 h at room temperature in the dark, dehydrated through graded ethanol and acetone, infiltrated with 812 resin, embedded, and then polymerized at 60 °C for 48 h. Ultrathin tissue sections (60–80 nm) were cut, stained with uranyl acetate and lead citrate, air-dried, and examined using TEM.

### 4.15. Iron Measurement

Ferrous (Fe^2+^) levels in mice cardiac tissue were ascertained by adhering to the protocol outlined by the Iron Assay Kit. The hearts of mice were initially perfused with PBS, followed by the excision of ventricular myocardial tissue. Equal masses of the tissue were homogenized to prepare protein lysates, and iron concentrations were subsequently calculated based on the tissue mass.

### 4.16. Serum CK-MB and LDH Measurement

Serum levels of CK-MB and LDH in mice were measured using commercial assay kits, as per the manufacturer’s instructions, with absorbance recorded at 593 nm.

### 4.17. Immunofluorescence (IF)

For H9C2 cardiomyocytes, cells were fixed with 4% paraformaldehyde at room temperature for 20 min, followed by permeabilization with 0.2% Triton X-100 for 10 min. The cells were then blocked with BSA for 1 h at room temperature. Next, the cell samples were incubated overnight with primary antibodies against GPX4 (1:200) and NEDD4L (1:200). After they were washed, fluorophore-conjugated secondary antibodies were added, and cell nuclei were stained with DAPI. Images were acquired using a confocal microscope.

For cardiac tissue, 5 μm thick tissue sections were subjected to antigen retrieval, followed by permeabilization, blocking, primary antibody incubation, secondary antibody incubation, and DAPI nuclear staining as described for cell immunofluorescence.

### 4.18. Immunoblots (IBs) and Immunoprecipitation (IP)

Mouse cardiac tissues were homogenized and sonicated in lysis buffer. The cells (H9C2 and HEK293T) were directly lysed in lysis buffer followed by sonication. Protein concentrations were determined using the BCA assay, and samples were adjusted to equalize protein content with RIPA buffer. Proteins were denatured by boiling them for 10 min. Equal amounts of protein from each sample were separated using SDS-polyacrylamide gel electrophoresis using a gel apparatus (Bio-Rad) and transferred onto a polyvinylidene fluoride (PVDF) membrane. The blotting membrane was blocked with 5% skim milk for 2 h at room temperature. The membrane was then incubated with the primary antibody overnight at 4 °C. Anti-mouse or anti-rabbit IgG antibodies were used as secondary antibodies and incubated for 2 h at room temperature. Signal detection was performed using an enhanced chemiluminescence (ECL) method.

For immunoprecipitation (IP), HEK293 cells, H9C2 cells, or cardiac tissues were lysed in 1× RIPA buffer supplemented with protease inhibitors. After centrifugation, the collected supernatants were incubated with specific IP antibodies at 4 °C for 1 h, followed by incubation with Protein A/G Plus-Agarose (sc-2003, Santa Cruz, CA, USA) at 4 °C for 6 h. The immunocomplexes were then washed five times with 1× RIPA buffer. After they were washed, the samples were resuspended in electrophoresis sample buffer and boiled to elute the immunoprecipitated fractions.

### 4.19. Statistical Analysis

Data from this study are presented as mean ± standard deviation (SD). Statistical analysis and data visualization were performed using GraphPad Prism version 9.0 (GraphPad Software, La Jolla, CA, USA). Comparisons between two groups were made using Student’s *t*-test, while analyses involving three or more groups were conducted using one-way analysis of variance (ANOVA). A *p*-value of less than 0.05 was considered to indicate statistical significance.

## 5. Conclusions

In this study, we elucidated the role of NEDD4L in cardiomyocytes under DOX treatment. Our findings identified a critical role for NEDD4L in promoting ferroptosis and apoptosis through the ubiquitination of GPX4 in DIC. This study provides new insights into the mechanisms underlying DIC and suggests that targeting NEDD4L may represent a potential therapeutic strategy for alleviating DIC.

## Data Availability

The data that support the findings of this study are available from the corresponding author upon reasonable request.

## Supplementary Materials

The following supporting information can be downloaded at: https://www.mdpi.com/article/10.3390/ijms26178201/s1.

## Abbreviations

The following abbreviations are used in this manuscript:

AAV9	Serotype 9 Adeno-associated Virus
CHX	Cycloheximide
CQ	Chloroquine
DIC	Doxorubicin-induced Cardiotoxicity
DOX	Doxorubicin
Fer-1	Ferrostatin-1
GPX4	Glutathione Peroxidase 4
LVEF	Left Ventricular Ejection Fraction
FS	Fractional Shortening
MDA	Malondialdehyde
NEDD4L	Neural Precursor Cell Expressed Developmentally Down-regulated Protein 4-like
ROS	Reactive Oxygen Species
Ub	Ubiquitin
