# Supplementary material for: NEDD4L-Mediated Ubiquitination of GPX4 Exacerbates Doxorubicin-Induced Cardiotoxicity
Source: Int J Mol Sci. 2025 Aug 23;26(17):8201. doi: 10.3390/ijms26178201 (PMC12428739; doi:10.3390/ijms26178201)
Supplement: Supplementary file 1 [file ijms-26-08201-s001.zip › ijms-3803560-supplementary.pdf]

Supplementary figures

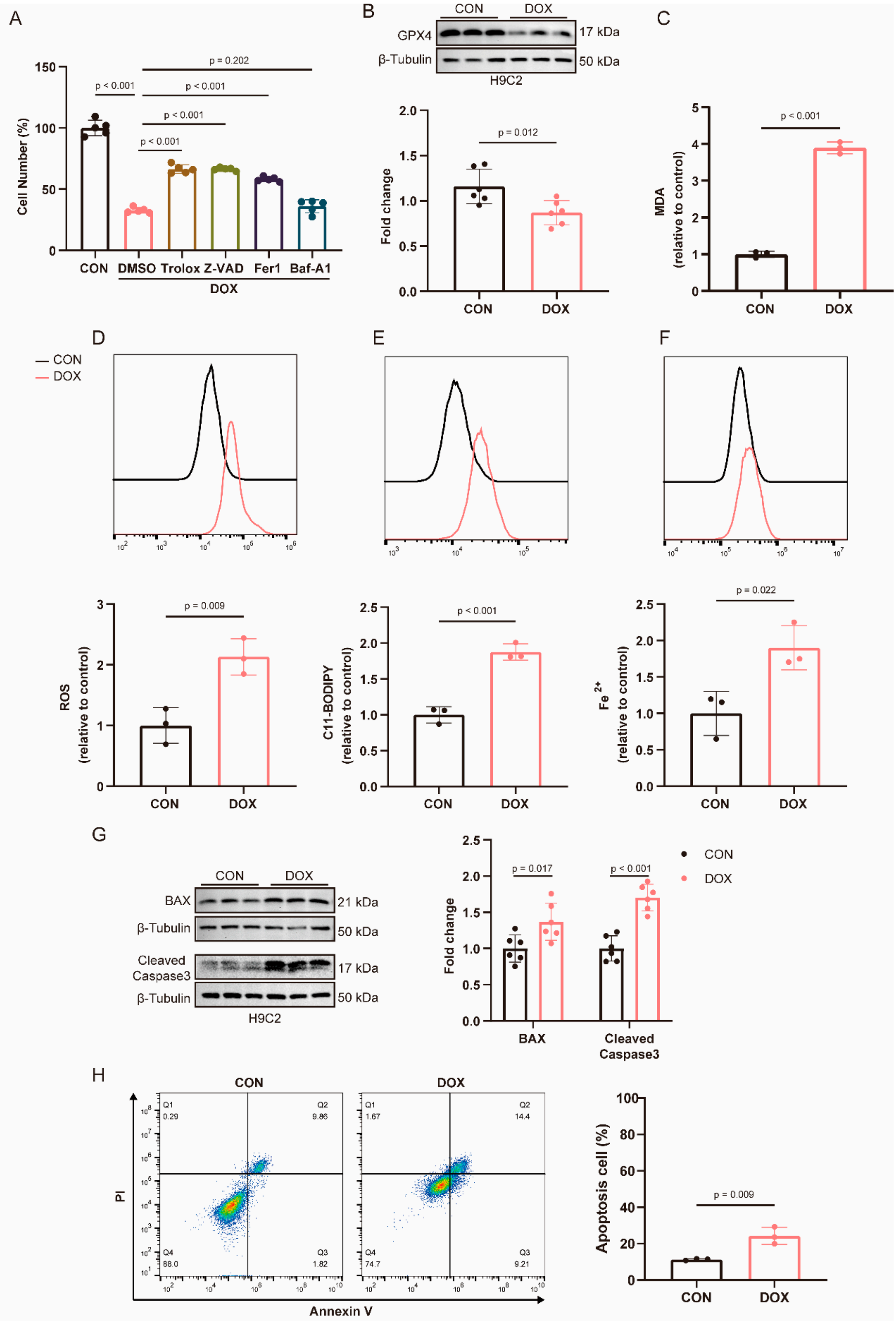

Figure S1. Doxorubicin (DOX) induces ferroptosis and apoptosis in cardiomyocytes.

A. Cell Counting Kit-8 (CCK-8) assessment of H9C2 cell response to DOX (2 μM)

following treatments with Trolox (100 μM), Z-VAD (10 μM), Fer-1 (2 μM), and Baf-A1 (1 nM) for 24 hours (*n* = 6).
B. Representative immunoblots and relative quantification of GPX4 in H9C2 cells (*n* = 6) treated with DOX (2 μM, 24 hours) or left untreated.
C. The malondialdehyde (MDA) content of H9C2 cells (*n* = 3) treated with DOX (2 μM, 24 hours) or left untreated.
D-F. Histogram and relative quantification of reactive oxygen species (ROS, C), lipid peroxide (D), and ferrous iron (Fe^{2+}, E) levels in H9C2 cells treated with DOX (2 μM, 24 hours) or left untreated, detected using the staining probe of DCFH-DA, C11-BODIPY, and FerroOrange (*n* = 3).
G. Representative immunoblots and relative quantification of proteins related to apoptosis in H9C2 cells (*n* = 6) treated with DOX (2 μM, 24 hours) or left untreated.
H. The effects of DOX treatment on cardiomyocyte apoptosis using flow cytometry analysis (*n* = 3).

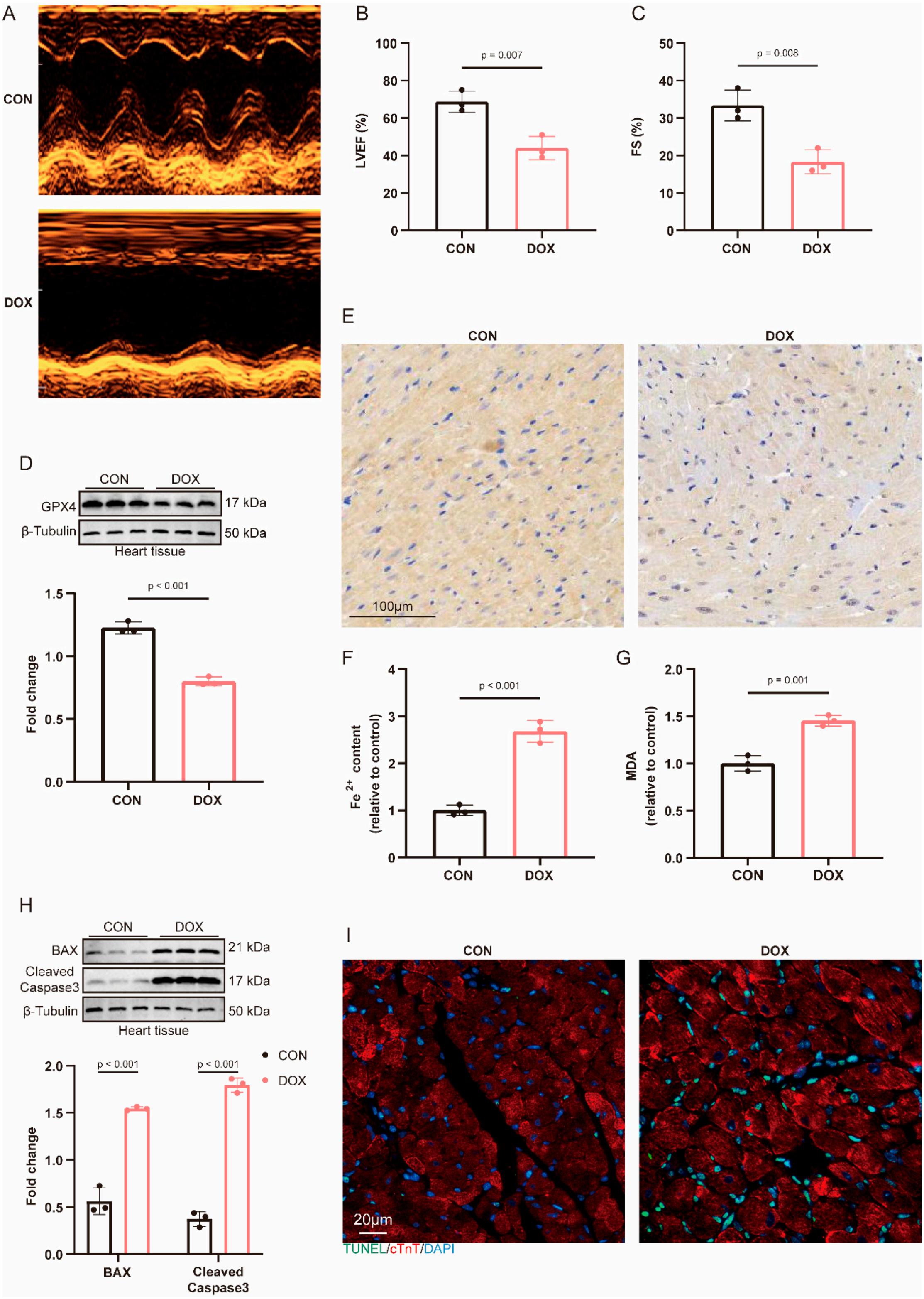

Figure S2. Doxorubicin (DOX) induced cardiotoxicity (DIC) characterized by the impairment of cardiac function, ferroptosis, and apoptosis.

A-C. Representative left ventricular M-mode echocardiography images (A) and echocardiographic assessment of left ventricular ejection fraction (LVEF, B) and fractional shortening (FS, C) in mice 7 days post-intraperitoneal saline or DOX (15 mg/kg) injection (n = 3).

D. Representative immunoblots and relative quantification of GPX4 in heart tissue of mice subjected to intraperitoneal injection of either saline or DOX (15 mg/kg) (n = 3).
E. Immunohistochemical analysis of GPX4 expression in heart tissues of mice subjected to intraperitoneal injection of either saline or DOX (15 mg/kg).
F-G. Relative quantification of myocardial iron content (Fe^{2+}, D) and malondialdehyde (MDA, E) levels in mice subjected to intraperitoneal injection of either saline or DOX (15 mg/kg) (n = 3).
H. Representative immunoblots and relative quantification of proteins related to apoptosis in heart tissues from mice subjected to intraperitoneal injection of either saline or DOX (15 mg/kg) (n = 3).
I. Terminal deoxynucleotidyl transferase-mediated dUTP nick end labeling (TUNEL) staining of heart tissues from mice subjected to intraperitoneal injection of either saline or DOX (15 mg/kg).

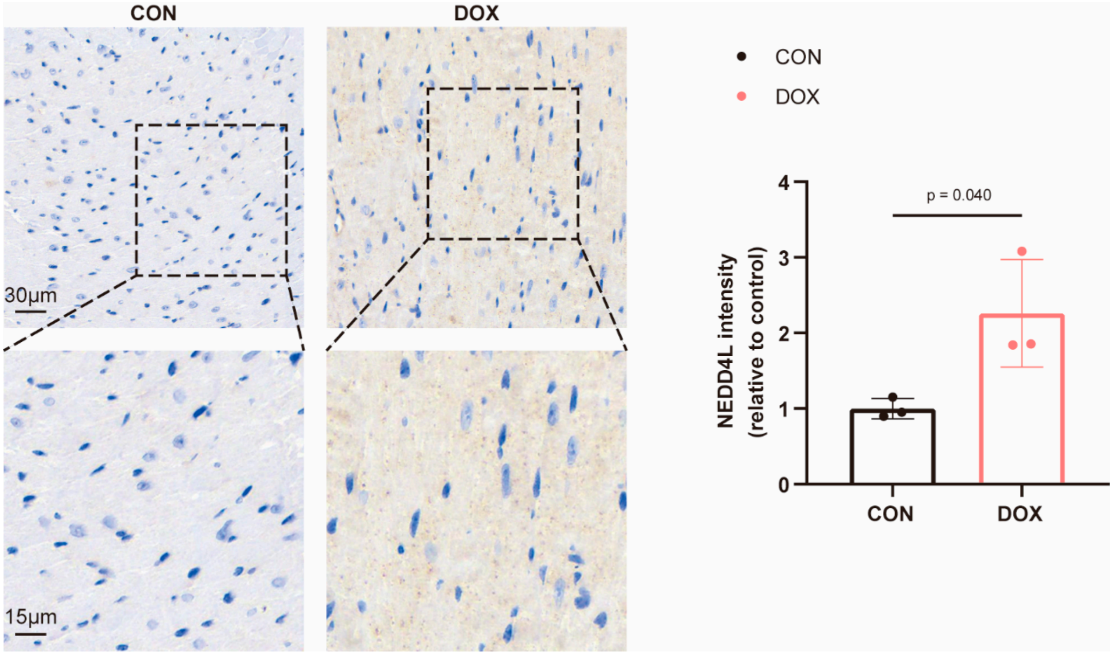

Figure S3. Immunohistochemical analysis and relative quantification of NEDD4L expression in heart tissues from mice subjected to intraperitoneal injection of either the vehicle control (saline) or doxorubicin (DOX) (*n* = 3)

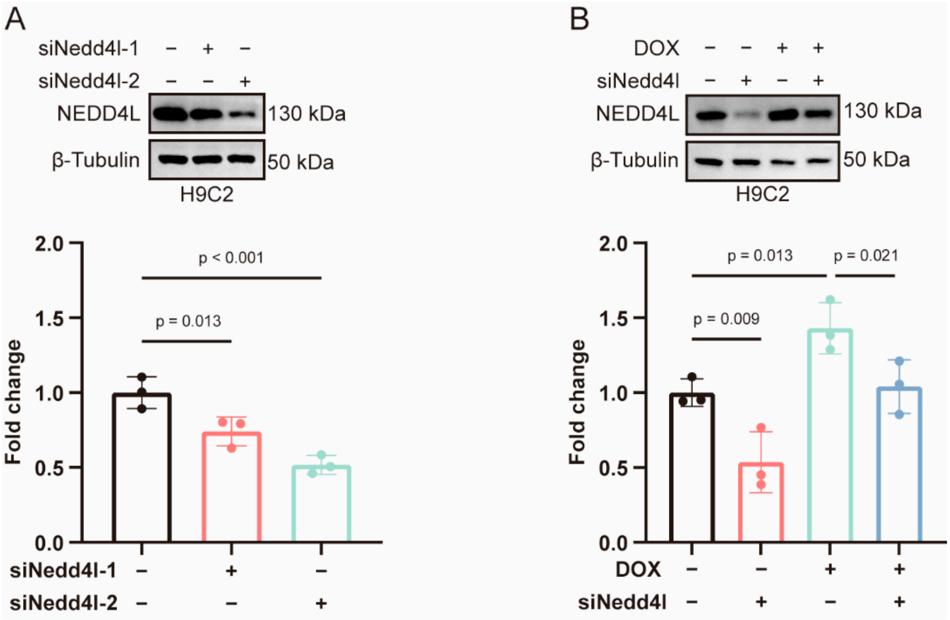

Figure S4. Validation of small interfering RNA targeting NEDD4L (siNedd4l) knockdown efficiency in H9C2 cells.

A. Representative immunoblots and relative quantification of NEDD4L in H9C2 cells following transfection with a non-targeting control (siNC), siNedd4l-1, or siNedd4l-2 (*n* = 3).

B. Representative immunoblots and relative quantification of NEDD4L in H9C2 cells following treatment with siNedd4l or siNC, and exposure to DOX (2 µM, 24 hours) (*n* = 3).

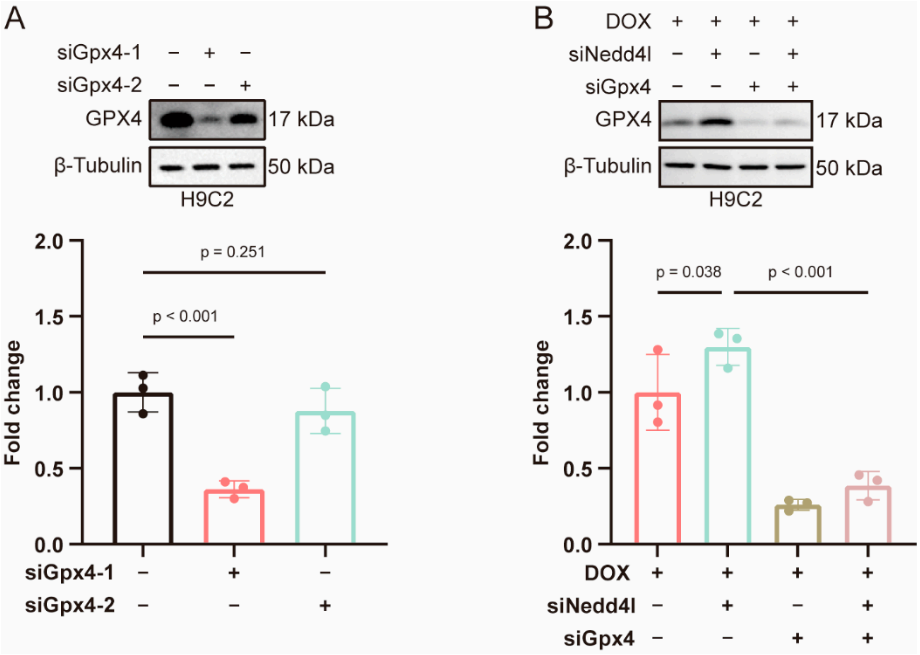

Figure S5. Knockdown of GPX4 in H9C2 cells assessed using Western blot analysis.

A. Representative immunoblots and relative quantification of GPX4 in H9C2 cells treated with a non-targeting control (siNC) or two different small interfering RNAs targeting GPX4 (siGpx4-1 and siGpx4-2) (*n* = 3).

B. Representative immunoblots and relative quantification of GPX4 in H9C2 cells with siNedd4l/siNC and siGpx4/siNC pretreatments followed by DOX (2 μM, 24 hours) intervention (*n* = 3).
